# Maternal thrombocytopenia is not predictive of neonatal thrombocytopenia: a single-center Irish study

**DOI:** 10.1016/j.rpth.2024.102622

**Published:** 2024-11-14

**Authors:** Ligia Nechifor, Daniel O’Reilly, John O’Loughlin, Fionnuala Ní Áinle, Naomi Mc Callion, Lyudmyla Zakharchenko

**Affiliations:** 1 Department of Paediatrics, The Rotunda Hospital, Dublin, Ireland; 2 Conway Sphere Research Group, School of Biomolecular and Biomedical Science, University College Dublin, Dublin, Ireland; 3 Department of Laboratory Medicine, The Rotunda Hospital, Dublin, Ireland; 4 Department of Haematology, The Rotunda Hospital, Dublin, Ireland; 5 Department of Paediatrics, Royal College of Surgeons, Dublin, Ireland; 6 School of Medicine, University College Dublin, Dublin, Ireland

**Keywords:** full blood count, neonatology, obstetrics, platelets, pregnancy, thrombocytopenia

## Abstract

**Background:**

Maternal thrombocytopenia during pregnancy is common. However, the relationship between maternal and neonatal thrombocytopenia is poorly understood.

**Objectives:**

We aimed to determine whether an association exists between platelet counts of neonates born to mothers with moderate-to-severe thrombocytopenia (<100 × 10^9^/L) and neonatal platelet counts.

**Methods:**

We identified records from 557 patients with moderate-to-severe thrombocytopenia (maternal platelet count <100 × 10^9^/L) and the 338 associated newborn charts from 2018 to 2022 in a single large maternity center. Pregnant people with a platelet count of <100 × 10^9^/L prior to delivery during present gestation were included. Any thrombocytopenia that occurred outside of pregnancy or in the postpartum period was excluded. A logistic regression was then generated to examine the association between maternal thrombocytopenia and neonatal thrombocytopenia. A receiver operating characteristic (ROC) curve was generated to assess accuracy of (i) lowest maternal platelet count and (ii) trimester of thrombocytopenia onset in predicting neonatal thrombocytopenia.

**Results:**

A total of 550 full blood count assessments were taken in neonates of pregnant people with thrombocytopenia. Sixteen neonates with clinically significant thrombocytopenia (platelet count <100 × 10^9^/L) were identified. A binomial logistic regression was fitted that demonstrated limited association between lowest maternal platelet count and trimester of onset of maternal thrombocytopenia and the development of neonatal thrombocytopenia. An ROC curve was generated to determine the accuracy of maternal platelet count at identifying neonatal thrombocytopenia. The coordinates of the best platelet count threshold for this dataset were then derived from the ROC curve and determined that a threshold of 77.5 × 10^9^/L maternal platelets offered the best accuracy.

**Conclusion:**

Neonatal full blood count assessment based on maternal platelet counts of <100 × 10^9^/L has a poor diagnostic yield with no statistically significant association in this cohort on logistic regression analysis. A lower threshold of 77.5 × 10^9^/L may be of higher clinical utility and improve laboratory and clinical workflow.

## Essentials

• A low platelet count (thrombocytopenia) is a common finding in pregnancy.
• Neonates from mothers with thrombocytopenia had their platelet count assessed in an Irish hospital.
• Maternal platelet count below 100 × 10^9^/L was not associated with neonatal thrombocytopenia.
• Maternal thrombocytopenia below 77 × 10^9^/L most accurately predicts neonatal thrombocytopenia.

## 1 Introduction

Thrombocytopenia is second only to anemia as the most common hematologic abnormality that occurs during pregnancy, affecting 9% to 12% of pregnant people [1]. While 70% to 80% of cases of thrombocytopenia represent incidental thrombocytopenia of pregnancy, or “gestational” thrombocytopenia, there is often concern among clinicians caring for the maternal-fetal dyad that a rarer but more significant cause, such as immune thrombocytopenia (ITP), might be responsible for the fall in platelet counts [2,3].

Thrombocytopenia is also common in the neonatal period, with 1% of neonates having a platelet count <150 × 10^9^/L [4]. There are numerous causes of neonatal thrombocytopenia, with placental insufficiency and infection being the most common. While the most common neonatal ITP is neonatal alloimmune thrombocytopenia, other ITPs caused by maternal antiplatelet antibodies also occur. Conditions such as ITP and antiphospholipid syndrome are associated with maternal thrombocytopenia and intracranial hemorrhage and are important to detect as they have consequences for neonatal monitoring after delivery [5].

Expert consensus exists regarding the assessment of platelet count in mothers with known ITP, but there is no current guidance on a threshold of maternal thrombocytopenia that should trigger the evaluation of the newborn’s platelet count [1,5,6]. In our institution, current local practice is that a neonatal platelet count is performed where maternal platelet counts reached <100 × 10^9^/L. A recent study investigating pregnant people with a low platelet count on admission to the delivery ward suggested limited correlation with the development of neonatal thrombocytopenia, but conclusions were restricted by lack of data from earlier in pregnancy and the relative rarity of neonatal thrombocytopenia in a predominantly healthy cohort of pregnant people (*n* = 218 with platelet counts <100 × 10^9^/L) [7].

The purpose of this study was to examine the accuracy of maternal platelet counts in predicting neonatal counts in pregnant people with moderate-to-severe thrombocytopenia (<100 × 10^9^/L). We also sought to establish a “best fit” platelet count threshold for neonatal investigations. Finally, we investigated how a change in the platelet threshold triggering investigation would impact the number of full blood count (FBC) assessments performed on neonates for this reason.

## 2 Methods

### 2.1 Study design

**Figure 1:**
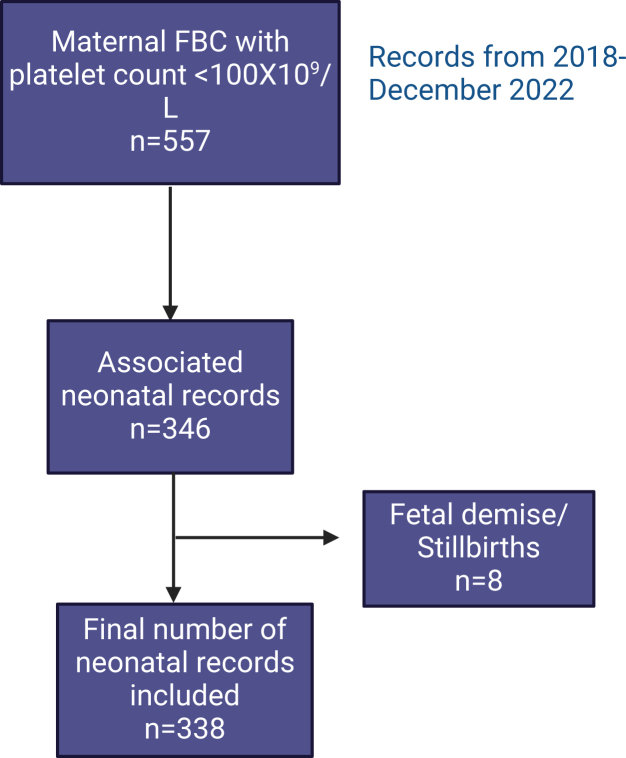
Flow diagram describing included patient data.

This was a single-site retrospective study in a tertiary maternity hospital (approximately 9000 deliveries per year), investigating a cohort of pregnant people whose pregnancy was complicated by moderate-to-severe thrombocytopenia from 2018 to 2022. The associated neonatal intensive care unit (NICU) is a tertiary unit that cares for neonates from 23 weeks corrected gestational age to term corrected gestational age. The NICU cares for inborn neonates who require any level of neonatal care as well as outborn infants requiring specialist neonatal care (very preterm, very low birth weight, hypoxic-ischemic encephalopathy). Pregnant people were identified using laboratory records of platelet counts <100 × 10^9^/L. As the hospital caters to both obstetric and gynecologic patients, each record was reviewed by researchers to establish whether thrombocytopenia occurred in the context of pregnancy. Additionally, each record was assessed to ensure more than a single FBC <100 × 10^9^/L was present to account for pre-analytical or pre-clinical error. When thrombocytopenia that occurred in the context of gynecologic procedures or early pregnancy loss were excluded, a total of *n* = 346 neonatal records for review remained, 8 of which were excluded due to late pregnancy loss or stillbirth. This left 338 neonatal records that were included in the study (Figure 1).

### 2.2 Data collection

Data on each maternal-neonatal dyad were collected. Maternal variables included lowest platelet count, trimester of onset of thrombocytopenia, maternal diagnosis of cause of thrombocytopenia and maternal treatments for thrombocytopenia. Neonatal variables included gestational age at birth, birth weight, sex, lowest maternal platelet count, number of FBC assessments performed on each infant, the reason each neonate had a FBC performed, lowest neonatal platelet count, and the occurrence of moderate-to-severe thrombocytopenia (defined as moderate: 50-100 × 10^9^/L, severe: 25-49 × 10^9^/L, very severe: <25 × 10^9^/L) in the newborn, major bleeds as defined in previous neonatal studies (intraventricular hemorrhage [IVH] grade 3 or above, any nonintraventricular intracranial hemorrhage, frank rectal or gastric bleeds or pulmonary hemorrhage with ventilation changes), or any interventions for neonatal thrombocytopenia [8]. Any diagnoses of the cause of thrombocytopenia in either mother or baby were also recorded.

### 2.3 Statistical analysis

All statistical analyses were performed on R statistical software (R foundation for statistical computing). Base R package “glm” was used to generate a binomial logistic regression. The “pROC” package was used to generate receiver operating characteristic (ROC) curves, determine “best” fit thresholds and to generate sensitivity and specificity to calculate the youden index [9].

### 2.4 Ethical approval

Study was reviewed and approved by the Rotunda Hospital research ethics committee (reference: RAG-2022-031).

## 3 Results

### 3.1 Clinical demographics of included maternal-neonatal dyads

We identified *n* = 557 individual patients in our maternity hospital who had thrombocytopenia (with platelet counts <100 × 10^9^/L) during this time period. Data were available on 338 maternal-neonatal dyads with clinical demographics summarized in Table 1. Two hundred ten neonates had a platelet count assessed, representing approximately 61% of included dyads. Five hundred fifty FBCs were performed in total. Sixteen neonates were identified with thrombocytopenia, with the majority of their mothers having a known diagnosis of ITP (*n* = 7). Half of the included neonates had a lowest platelet count of 51-100 × 10^9^/L and were monitored for further reductions in counts without specific intervention (Table 2). Gestational thrombocytopenia was diagnosed in 4 of the maternal-neonatal dyads with 3 neonates having a transient thrombocytopenia, which resolved without intervention and 1 neonate having nonimmune hydrops as a primary diagnosis. A single family with an autosomal dominant mutation known to cause thrombocytopenia without bleeding had 2 pregnancies in the time period, affecting the neonates on both occasions.

**Table 1:** Clinical demographics of included maternal-neonatal dyads.

	Totals (*n* = 338)	Neonates without thrombocytopenia <100 × 10^9^/L (*n* = 322)	Neonates with thrombocytopenia <100 × 10^9^/L (*n* = 16)
Gestational age at birth, wk, median (IQR)	39 (37+4-40)	39 (37+4-40)	39+3 (36+5-39+5)
Birth weight, g, median (IQR)	3330 (2850-3750)	3320 (2835-3738)	3600 (3100-3818)
Sex (% male)	198 (58.5%)	191 (59%)	7 (43.8%)
Lowest maternal platelet counts (×10^9^/L), median (IQR)	87 (76-94)	88 (77-94)	72 (57-83)
Trimester of maternal thrombocytopenia (%)	First: 28 (8.2%) Second: 28 (8.2%) Third: 282 (83.4%)	First: 24 (7.5%) Second: 25 (7.7%) Third: 273 (85%)	First: 4 (25%) Second: 3 (18.8%) Third: 9 (56.3%)
Maternal diagnosis, *n* (%)	Gestational thrombocytopenia *n* = 197 (58.2%) ITP *n* = 30 (8.8%) Preeclampsia/HELLP *n* = 67 (19.8%) Non-ITP immune thrombocytopenia *n* = 7 (2%) Infection *n* = 6 (1.7%) Chemotherapy *n* = 3 (0.08%) Familial/congenital thrombocytopenia *n* = 5 (1.5%) Other diagnoses *n* = 23 (6.8%)	Gestational thrombocytopenia *n* = 193 (59.9%) ITP *n* = 23 (7.1%) Preeclampsia/HELLP *n* = 64 (19.9%) Non-ITP immune thrombocytopenia *n* = 7 (2.2%) Infection *n* = 6 (1.9%) Chemotherapy *n* = 3 (0.9%) Familial/congenital thrombocytopenia *n* = 3 (0.9%) Other diagnoses *n* = 23 (7.1%)	Gestational thrombocytopenia *n* = 4 (25%) ITP *n* = 7 (43.7%) Preeclampsia/HELLP *n* = 3 (18.7%) Familial/congenital thrombocytopenia *n* = 2 (12.5%)
Maternal treatment	IVIG *n* = 15 (4.4%) Steroids *n* = 17 (5%) Transfusion *n* = 17 (5%)	IVIG *n* = 11 (3.4%) Steroids *n* = 16 (5%) Transfusions *n* = 17 (5.3%)	IVIG *n* = 4 (25%) Steroids *n* = 1 (6.25%)
Lowest neonatal platelet count (×10^9^/L), median (IQR)	234 (180-276)	243 (196-280)	51 (34-65)
Neonatal treatment	Transfusion *n* = 4 (1.1%) IVIG *n* = 4 (1.1%)	NA	Transfusion *n* = 4 (25%) IVIG *n* = 4 (25%)
Neonatal major hemorrhage (%)	IVH grade 3 or more *n* = 4 (1.1%) Subdural hemorrhage *n* = 2 (0.6%)		IVH grade 3 or more *n* = 4 (25%) Subdural hemorrhage *n* = 2 (12.5%)
<32 wk cGA (%)	17 (5%)	14 (4.3%)	3 (18.7%)
<1500 g bodyweight (%)	15 (4.4%)	13 (4%)	2 (12.5%)

cGA, corrected gestational age; HELLP, hemolysis, elevated liver enzymes, and low platelets; ITP, immune thrombocytopenia; IVH, intraventricular hemorrhage; IVIG, intravenous immunoglobulin; NA, not applicable.

**Table 2:** Grade of thrombocytopenia in observed cohort with platelet counts of <100 × 10^9^/L (*n* = 16).

Grade of thrombocytopenia	Number of neonates (%)
Moderate (50-100 × 10^9^/L)	8 (50%)
Severe (25-49 × 10^9^/L)	6 (37.5%)
Very severe (<25 × 10^9^/L)	2 (12.5%)

### 3.2 Maternal moderate-to-severe thrombocytopenia is a poor predictor of moderate-to-severe neonatal thrombocytopenia

To assess whether low maternal platelet count predicted the development of neonatal thrombocytopenia, a binomial logistic regression model was fitted with the development of neonatal thrombocytopenia (<100 × 10^9^/L) as the dependent variable, and maternal lowest recorded platelet count and trimester of diagnosis as the independent variables. There was no association with lowest maternal platelet count (*p* = .11; odds ratio [OR], 0.98; 95% CI, 0.95-1) or diagnosis of maternal thrombocytopenia in the first (*p* = .11; OR, 3.05; 95% CI, 0.67-11.7) or second trimester (*p* = .08; OR, 3.36; 95% CI, 0.7-12.19) with the eventual diagnosis of neonatal thrombocytopenia.

**Figure 2:**
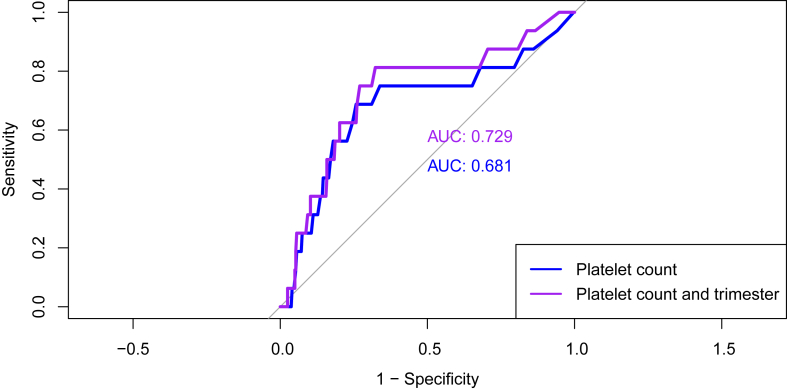
Receiver operating characteristic curves demonstrating accuracy of platelet count alone (blue) and platelet count and trimester of diagnosis (purple) in identifying neonates with thrombocytopenia. Associated area under the curve is also displayed.

An ROC curve was then fit using the data to establish the accuracy of using both maternal lowest platelet count and trimester of diagnosis to predict eventual neonatal thrombocytopenia (Figure 2). The areas under the curve of ROC curves fitted to (i) maternal lowest platelet counts and (ii) maternal lowest platelet counts and earlier trimester of thrombocytopenia onset were 0.68 and 0.73, respectively, suggesting limited discriminative ability.

To further examine the maternal platelet count that provided maximal accuracy, a “best fit” was performed on the ROC curve that suggested a threshold value of 77.5 × 10^9^/L, representing a sensitivity of 68% and a specificity of 74% in identification of neonatal thrombocytopenia <100 × 10^9^/L.

### 3.3 Number needed to diagnose neonatal thrombocytopenia based on maternal moderate-to-severe thrombocytopenia

In our cohort 550 FBC assessments were taken to identify 16 neonates with moderate-to-severe thrombocytopenia, so only 3% of tests resulted in diagnosis of relevant thrombocytopenia in neonates born to mothers with moderate-to-severe thrombocytopenia. To determine the number of assessments needed to successfully diagnose thrombocytopenia, we generated number needed to diagnose (NND) at a threshold of 100 × 10^9^/L and at the “optimal” threshold of 77.5 × 10^9^/L based on the previously described method of the inverse of youden index [10]. This produced an NND of 100 based on a maternal platelet count of 97.5 × 10^9^/L (as a youden index above this value is 0) and an NND of 2.4 based on a maternal platelet count of 77.5 × 10^9^/L.

In this cohort lowering the threshold to 77.5 × 10^9^/L would have reduced the number of FBCs performed by 270 investigations (49%), representing a substantial reduction in both clinical and laboratory workload. Of the neonatal cohorts who were identified as having thrombocytopenia, 5 neonates would have been “missed” using this new threshold. However, of these, 3 had only moderate low counts <100 × 10^9^/L (77, 94, and 98 × 10^9^/L) and were born to mothers with gestational thrombocytopenia with both maternal and neonatal counts recovering spontaneously, 1 had nonimmune hydrops fetalis that would have triggered investigation irrespective of maternal platelet count and 1 was extremely premature at 25 weeks corrected gestational age, where FBCs are performed as part of routine clinical care in the first few days of life, hence no neonates of concern would have been missed with this approach. Notably no neonate whose mother was affected by ITP would have been missed through reducing the platelet threshold in this cohort.

## 4 Discussion

This retrospective study of a large tertiary neonatal unit aimed to examine the diagnostic utility of FBC assessments in neonates born to mothers with moderate-to-severe thrombocytopenia. While some experts and prior studies have suggested that a maternal platelet count of <100 × 10^9^/L should prompt neonatal investigation, these data favor a lower threshold of 77.5 × 10^9^/L. This likely reflects that the pathophysiology of milder maternal thrombocytopenia is overwhelmingly gestational thrombocytopenia as opposed to immune mediated. It is recognized in the literature that gestational thrombocytopenia can result in platelet counts as low as 80 × 10^9^/L in the third trimester, and as the maternal platelet counts in this study are predominately from this trimester (83.4%) they may represent a normal variation in the late pregnancy population [2]. It is also important to note the overrepresentation of known ITP in pregnant people who delivered neonates with thrombocytopenia and the fact that all of these individuals had platelet counts below the suggested “optimum” threshold for neonatal investigation [5]. However, our overall incidence of 8.8% is not out of keeping with other published cohorts examining pregnant people with thrombocytopenia where the ITP incidence varies from 3% to 28% [11,12].

This study contributes to the existing literature on maternal thrombocytopenia and subsequent neonatal thrombocytopenia in numerous ways. First, it examines a large cohort of pregnant people with moderate-to-severe thrombocytopenia due to a variety of causes in a large tertiary maternity center. Second, it provides reassurance for pregnant people who have gestational thrombocytopenia in the third trimester that there is a low risk of clinically meaningful thrombocytopenia in their baby. Third, by reducing the threshold where FBC assessments are performed, it is possible to substantially reduce neonatal phlebotomy in what is largely a healthy and term population at low risk. While we identified a “best fit” maternal platelet count of 77.5 × 10^9^/L, this still remains a poor discriminator in identifying neonatal thrombocytopenia. A multifactorial approach taking into account other variables including presence/severity of ITP, thrombocytopenia in previous pregnancies and gestational age at time of onset would likely be a better predictor but would lack the parsimony of maternal platelet counts in what represents a largely healthy population.

Limitations to this study include the single site, retrospective nature of the cohort, however the large numbers of pregnant people and infants included support the likely validity in a wider population. There were a number of neonates in whom an FBC was not performed despite maternal thrombocytopenia <100 × 10^9^/L, which may reflect clinical decision making in a well-appearing neonatal cohort with no symptoms of bleeding or petechiae. As we analyzed maternal nadir values or lowest platelet values, a number of maternal platelet counts may have recovered by delivery and therefore neonates may not have been identified as born to a pregnant person with thrombocytopenia. We also identified only a small cohort of pregnant people whose thrombocytopenia was identified in the first or second trimesters (*n* = 28 in both groups). While inclusion of gestation of onset led to only a modest improvement in our model’s ability to predict neonatal thrombocytopenia, the overall small numbers limit conclusions that can be made in these patients especially given the higher incidence of autoimmune causes of thrombocytopenia in this cohort. Ethnicity is not reliably recorded in our institution on booking so these data are missing from our cohort but may have an effect on incidence of conditions such as antiphospholipid syndrome or systemic lupus erythematosus.

In conclusion, a lower maternal platelet threshold for assessing neonates for thrombocytopenia may be more accurate in detecting neonates with moderate-to-severe thrombocytopenia. This represents a parsimonious tool to reduce both the clinical and laboratory burden of testing as well as avoid unnecessary painful phlebotomy in healthy neonates.
